# Comparison between low molecular weight heparin and apixaban (direct oral anticoagulant) in the prophylaxis against venous thromboembolism after laparoscopic sleeve gastrectomy

**DOI:** 10.1007/s11695-025-07721-y

**Published:** 2025-02-08

**Authors:** Ahmed Abdelsalam, Michael Fikry, Ahmed Fahmy, Tarek Hegazy, Afaf Hamdy, Ahmed Refaat, Ahmed Elansary

**Affiliations:** 1 General Surgery Department, Faculty of Medicine, Cairo University, Giza, Egypt; 2 Diagnostic and Interventional Radiology Department, Faculty of Medicine, Cairo University, Giza, Egypt

**Keywords:** Bariatric surgery, Venous thromboembolic complications, Direct oral anticoagulants, Apixaban

## Abstract

**Background:**

Like any major operation, sleeve gastrectomy (SG) has its reported postoperative complications. Among them are venous thromboembolic complications (VTE) that may predispose to mortality. Despite the proven efficacy of the traditional anticoagulants, such as low molecular weight heparins (LMWHs) for VTE management, they have their limitations. Direct oral anticoagulants (DOACs) have been currently adopted for the management of VTE. We conducted this study to evaluate the efficacy and safety of apixaban against VTE after laparoscopic sleeve gastrectomy in comparison with LMWH.

**Methods:**

This was a randomized controlled trial that included 100 adult patients who underwent SG and received LMWH (Group A) or apixaban (Group B) for VTE prophylaxis. We recorded and analyzed the postoperative events up to the 30th day after surgery.

**Results:**

This study included Group A (n = 50) and Group B (n = 50). No VTE occurred in either group (0%). Postoperative bleeding was encountered in one patient of each group (2%). The follow-up venous Doppler study was unremarkable in the two groups.

**Conclusion:**

Apixaban was shown to be comparable to LMWH for the prevention of VTE after LSG with similar efficacy and safety making it a promising alternative to LMWH in patients undergoing bariatric surgery.

## Introduction

Obesity has become a global epidemic threatening human health, with reports estimating that, if the current obesity trend persists, obesity will be prevalent among more than one billion individuals by 2025, of whom 177 million will have severe obesity [1].

Bariatric surgery is currently established as the most definite, long-standing management of severe obesity [2]. In the modern era, sleeve gastrectomy (SG) has been one of the most popular bariatric procedures, attributing to its efficacy as well as technical ease and safety compared to other procedures [3]. It has been announced that SG is the most performed bariatric procedure since 2014 [4].

Like any major operation, SG has its reported postoperative complications. Among them are venous thromboembolic complications (VTE) that have been reported to occur after bariatric procedures, including SG, at a rate ranging from 0.3% to 2.4% [5–7]. Obesity itself is an established risk factor for VTE [8]. Pulmonary embolism (PE), one of the VTE complications, accounts for 40% of the 30-day postoperative mortality after bariatric surgery [9–12].

Despite the proven efficacy of the traditional anticoagulant drugs, such as vitamin K antagonists and low molecular weight heparins (LMWHs) for VTE management, they are limited by the parenteral administration and the requirement to be continuously monitored by the international normalized ratio (INR) [13].

Direct oral anticoagulants (DOACs) have been currently adopted for the management of VTE. Some of these medications, including apixaban, endoxaban, and rivaroxaban, are selective and reversible direct inhibitors of factor Xa, thus inhibiting the formation of fibrin-antagonists. They have several advantages over the traditionally used anticoagulants, including the faster onset of action, non-requirement to laboratory monitoring, higher efficacy in the prophylaxis against stroke, less incidence of major bleeding complications, and less incidence of drug-food interaction [13–16].

However, there is scarce research investigating the outcome of the routine implementation of apixaban as for the postbariatric surgery VTE prophylaxis [17–19], with all studies addressing this issue being retrospective studies. Furthermore, as far as we could reach in the literature, head-to-head comparisons between apixaban and LMWH in this clinical setting do not exist. This study was conducted to assess the efficacy and safety of apixaban against VTE after laparoscopic sleeve gastrectomy in comparison with LMWH.

## Patients and Methods

This was a prospective randomized controlled study that involved patients who were recruited for laparoscopic sleeve gastrectomy (LSG) at our institution during the period from March 2022 till January 2024. The research ethics committee approval (No. MD-194–2022) was obtained before the initiation of the study that was conducted in adherence to the Declaration of Helsinki. The trial was registered in the Pan African Clinical Trial Registry (PACTR202410819654281).

Consecutive adult patients who were recruited for LSG after ensuring being fit for bariatric surgery as per the internationally recognized criteria [20–22] were included in the study.

The initially included patients underwent history taking, a full general assessment including anthropometric measures, routine preoperative investigations including coagulation and hormonal (thyroid functions and serum cortisol morning level assay to exclude the medical causes of obesity) profiles, pelvi-abdominal ultrasonography, echocardiography, and a Doppler study on mesenteric, portal, splenic, and both lower limb venous systems.

Patients with normal preoperative serum levels of human coagulation factors, normal echocardiography, and normal venous Doppler studies were eligible for the current study. Patients with risk factors for VTE beyond obesity, including those with prolonged immobility, a history of previous VTE, recent surgery or trauma, cancer, hormonal therapy, chronic medical conditions, smoking, or advanced age as well as women in the postpartum period, were excluded from the study. Patients who were not tolerating oral fluids day 1 postoperatively (persistent vomiting), patients presented with early (day 0 or 1) postoperative bleeding, and those who missed the follow-up Doppler study were also excluded. Informed written consents were obtained from all the included patients.

### Randomization

The randomization of patients was performed using a computer-based random distribution system. Patients were allocated into Group A, including patients who received LMWH for VTE prophylaxis, and Group B, including patients who received apixaban.

### Intervention

In both groups, the surgery was done as standardized. All patients received general anesthesia. Compression stockings were applied on the patient’s lower legs. After creation of pneumoperitoneum, laparoscopic sleeve gastrectomy was done through the same technique using a 36-F calibration tube (Bougie). All patients underwent a drain insertion.

Postoperative instructions regarding proton pump inhibitors administration and diet regimens were prescribed, and patients in both groups were encouraged of early mobility.

### Group A

Patients in Group A received postoperative LMWH from day 1 to day 15 at a dose of 1 mg/kg/day, not exceeding 120 mg/day.

### Group B

Patients in Group B received postoperative apixaban at a dose of 2.5 mg every 12 h from day 1 to day 15 postoperatively.

### Follow-up

All patients received routine postoperative monitoring. A follow-up venous Doppler study was performed on day 15 postoperatively with the same device and the same operator. The patients’ baseline, operative, and postoperative data till day 30 were collected and recorded.

### Study outcomes

The study outcomes were the rate of VTE events as well as bleeding complications till the day 30 after surgery.

### Statistical analysis

The study’s data were analyzed using version 26.0 of the SPSS software (Armonk, NY: IBM Corp). Data were expressed as numbers and percents, or mean ± standard deviation, and compared using the chi-square test/z-test for proportion or an independent t-test accordingly. The significance of the obtained results was judged at the 5% level.

## Results

**Fig. 1:**
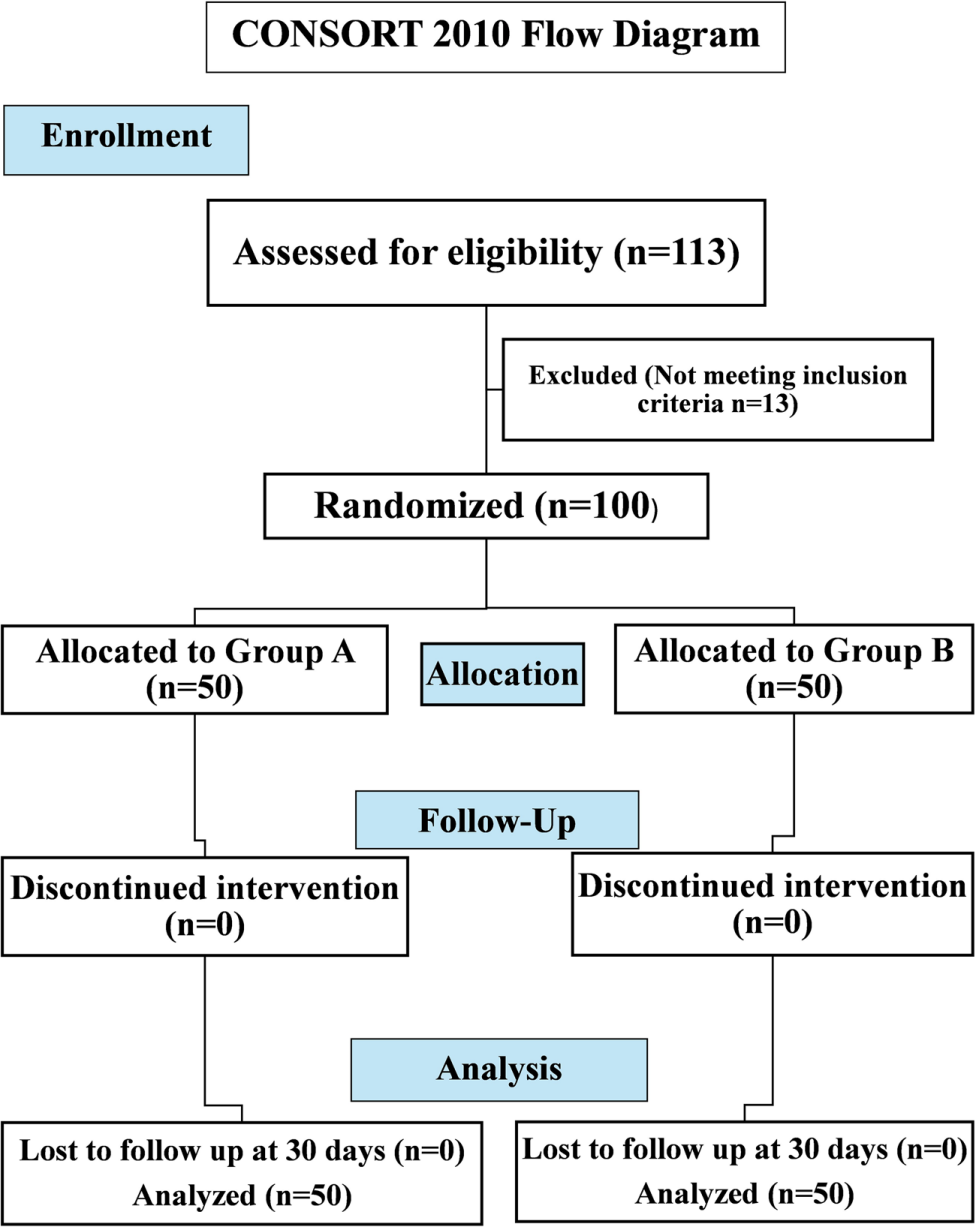
Allocation of group A and B in the study

This study finally included 100 patients who underwent LSG and were recruited to Group A (n = 50) and Group B (n = 50) during the period from March 2022 till January 2024, with no patients’ dropping out till day 30 (Fig. 1 -according to consort 2010 flow diagram-). The patients’ age ranged from 18 to 59 years old, with a mean of 36 ± 10.17 years, with matched age in the two groups (mean of 35.08 ± 9.46 in Group A and 36.90 ± 10.86 in Group B, p = 0.375). There was comparable female predominance in the two groups, constituting 78.0% of Group A and 86.0% of Group B (p = 0.298) (Table 1).

**Table 1:** Demographic and Baseline Characteristics

Characteristic	Group A (n = 50)	Group B (n = 50)	p-Value
Age (years)	35.08 ± 9.46	36.90 ± 10.86	0.375
Gender Distribution			
Female (%)	38 (78.0%)	43 (86.0%)	0.298
Male (%)	12 (22.0%)	7 (14.0%)	
Baseline Weight (kg)	130.72 ± 16.71	125.02 ± 20.89	0.135
Baseline BMI (kg/m^2^)	49.12 ± 5.22	47.37 ± 7.19	0.161
Obesity-Associated Complications			
Yes	12 (24.0%)	18 (36.0%)	0.190
No	38 (76.0%)	32 (64.0%)	
Dyslipidemia (%)	38 (76.0%)	31 (82.0%)	0.459
Hypertension (%)	8 (16.0%)	10 (20.0%)	0.603
Type 2 Diabetes Mellitus (%)	5 (10.0%)	10 (20.0%)	0.162
hypothyroidism	1 (2.0%)	1 (2.0%)	–
Ischemic heart disease	1 (2.0%)	1 (2.0%)	–
Bronchial asthma	1 (2.0%)	1 (2.0%)	–

The baseline weight was 130.72 ± 16.71 kg in Group A and 125.02 ± 20.89 kg in Group B and the mean baseline BMI was 49.12 ± 5.22 kg/m2 and 47.37 ± 7.19 kg/m2 in the two groups, respectively, with no statistically significant differences in either weight (p = 0.135) or BMI (p = 0.161) (Table 1).

Obesity-associated medical complications were prevalent in 24% of patients in Group A (n = 12) and 36% of patients in Group B (n = 18), with no statistically significant difference (p = 0.190). The most common complication in the two groups was dyslipidemia (76% of Group A and 82% of Group B), followed by hypertension (16% of Group A and 20% of Group B), and type 2 diabetes mellitus (10% of Group A and 20% of Group B), with no statistically significant differences (p = 0.459, 0.603, and 0.162, respectively) (Table 1).

The mean operative times were 1.94 ± 0.48 h and 1.95 ± 0.60 h, with no statistical difference between the two groups. The mean lengths of hospital stay were 2.04 ± 0.28 days and 2.16 ± 0.82 days in the two groups with no statistically significant differences (p = 0.927 and 0.330, respectively) (Table 2).

**Table 2:** Operative Details and Postoperative Outcomes

Characteristic	Group A (n = 50)	Group B (n = 50)	p-Value
Mean Operative Time (hours)	1.94 ± 0.48	1.95 ± 0.60	0.927
Mean Length of Hospital Stay (days)	2.04 ± 0.28	2.16 ± 0.82	0.330
Readmission Rate	1 (2.0%)	1 (2.0%)	1.000
Postoperative Complications	2 (4.0%)	5 (10.0%)	0.238
Venous Thromboembolism	0 (0.0%)	0 (0.0%)	–
Postoperative Bleeding	1 (2.0%)	1 (2.0%)	–
Mortality	0 (0.0%)	0 (0.0%)	–

In Group A, one patient was readmitted after discharge due to fever with clinical suspicion of a minor leak. The patient was managed conservatively and discharged uneventfully. In Group B, one patient was readmitted due to fever and cough. Clinical examination revealed a chest infection and mild pleural effusion. The patient was managed conservatively. The readmission rate was the same in the two groups (n = 1; 2% for each).

As for the efficacy and safety of the used drugs, no VTE occurred in either group (0%). Postoperative bleeding was encountered in one patient of each group (2%). The follow-up venous Doppler study was unremarkable in the two groups (Table 2).

The incidence of early postoperative adverse events was 4% in Group A (n = 2). These were postoperative bleeding in one patient (2%) and minor leaks in another patient (2%). In Group B, the incidence of postoperative adverse events was 10% (n = 5). These were fever in four patients (two patients had port site infection and two patients had chest infection), and postoperative bleeding in the last patient. The difference between the two groups in early complications wasn’t statistically significant (p = 0.238). No mortality was encountered in either group (Table 2).

## Discussion

Low-molecular-weight heparin has traditionally been the cornerstone of VTE prophylaxis in surgical patients, including those undergoing bariatric procedures. Its efficacy in reducing the incidence of VTE is well documented, and it is widely used due to its predictable pharmacokinetics and efficacy. However, direct oral anticoagulants, such as apixaban, have emerged as potential alternatives to LMWH, offering the convenience of oral administration and a favorable safety profile in various settings. Despite this, the use of DOACs in the bariatric surgery population remains a topic of debate, with concerns regarding their absorption, efficacy, and safety in this unique patient group.

The present study showed that apixaban was as effective and as safe as LMWH in the prophylaxis against VTE, with no VTE events encountered and a similar low rate of bleeding reported in either group. Postoperative bleeding occurred at a rate of 2% in the current study; however, the bleeding is likely not related to anticoagulation as it occurred on day 2 postoperatively. Therefore, it was likely attributed to reactionary hemorrhage rather than the effect of anticoagulation.

Although direct studies specifically targeting bariatric surgery are limited, related research in high-risk surgical populations provides valuable comparative data.

One meta-analysis study conducted by Caldeira et al. [23], albeit focused on patients undergoing major orthopedic surgery, found that apixaban offers a comparable, if not superior, VTE prevention compared to LMWH. The study highlighted that apixaban significantly reduced the incidence of VTE events and overall mortality without increasing the risk of major bleeding when compared to LMWH. Another study by Koo [24] focused on postoperative thromboprophylaxis in hip arthroplasty and concluded that new oral anticoagulants, including apixaban, were more effective than LMWH in reducing VTE risk without increasing bleeding complications. Additionally, Cohen et al. [25], in their retrospective analysis of VTE patients with active cancer, demonstrated that apixaban had lower incidences of risk of bleeding and recurrent VTE compared to LMWH, indicating its potential as a safer alternative in high-risk populations. These findings suggest that apixaban could be a viable alternative to LMWH in patients undergoing laparoscopic sleeve gastrectomy, offering similar protective benefits with a comparable safety profile.

In the realm of bariatric surgery, although specific studies directly comparing DOACs and LMWH in bariatric surgery are scarce, the existing research provides a foundation for understanding their relative advantages. A study conducted by Guzman-Pruneda et al. [18] demonstrated that patients who received apixaban at a dose of 2.5 mg twice daily for 30 days post-discharge experienced no VTE events, whereas the control group had a 0.5% incidence of VTEs. However, the study also noted a slightly higher rate of bleeding events in the apixaban group compared to the non-apixaban group (0.5% vs. 0.3%). Despite these variations, the differences were not statistically significant, and most bleeding incidents were minor, requiring readmission for observation rather than invasive interventions.

A comprehensive multicenter study was performed by Surve et al. [17] to investigate the use of apixaban as a thromboprophylactic agent in a large cohort of post-bariatric surgery patients, including 5017 patients who underwent various bariatric procedures and were subsequently treated with apixaban at a dose of 2.5 mg twice daily for 30 days, starting on the third postoperative day. The low incidence of thromboembolic events in patients who adhered to the apixaban regimen in the described study suggests that this drug is highly effective in preventing potentially fatal complications like porto-venous thrombosis and PE. The relatively low rate of side effects, coupled with the fact that most were mild, further supports the safety profile of apixaban in this context. Similar conclusions were reached by other studies assessing the role of DOACs in VTE prophylaxis after bariatric surgery [19, 26].

Duration of surgery is a well-known factor influencing the risk of VTE [27]. In this study, the operative times were comparable between the groups, minimizing any confounding impact of this variable on the outcomes.

It is worth noting that this study was conducted during and just after COVID-19 pandemic, which necessitated strict perioperative protocols to ensure patient safety. As part of routine standardization for elective surgeries during this time, patients with a current or recent history of COVID-19 or upper respiratory tract infections did not undergo surgery, in accordance with established guidelines and departmental policies [28, 29]. This standardization was implemented to mitigate the elevated risks associated with perioperative complications, including thromboembolic events, which are known to be heightened in the presence of active or recent COVID-19 infection.

We acknowledge the potential relevance of COVID-19-related hypercoagulability in the context of VTE prevention. However, recent studies have indicated that the risk of VTE among non-hospitalized patients with a history of COVID-19 is relatively low [30, 31]. By adhering to these routine protocols, we believe that the study results were not confounded by this variable.

Concerning the overall early postoperative adverse events, this was higher in the apixaban group (10%) compared to the LMWH group (4%), although this difference was not statistically significant. The complications in the apixaban group included port site infections, chest infections, and postoperative bleeding. The LMWH group, in contrast, had a lower overall complication rate, with only two reported events: postoperative bleeding and minor leak. The trend towards increased risk of certain adverse events with apixaban could be attributed to the higher rate of preoperative obesity-associated medical complications in the apixaban group.

Overall, this study emphasized the efficacy and safety of apixaban use after bariatric surgery for VTE prophylaxis. In the context of bariatric surgery, where patient compliance and ease of administration are crucial, the oral route of DOACs offers a distinct advantage over the injectable LMWH. Moreover, the rapid onset of action and the availability of antidotes for DOACs, as well as the cheaper price, further enhance their appeal, especially in a surgical setting where quick intervention is often required in cases of bleeding.

The current work is limited by the relatively small sample size and being a single-center study. However, to the best of our knowledge, this is the first study comparing apixaban with traditional LMWH for VTE thromboprophylaxis after bariatric surgery. Furthermore, the study was performed in the RCT design, which is the highest level of evidence in clinical research. This strengthens the validity of the findings. Despite the limitations, the results provide valuable insights that can inform clinical practice and pave the way for larger, multicenter trials to further validate these findings and potentially establish new standards for postoperative care in bariatric surgery patients.

## Conclusion

Apixaban was shown to be comparable to LMWH for the prevention of VTE after LSG with similar efficacy and safety making it a promising alternative to LMWH in patients undergoing bariatric surgery.

## Data Availability

Datasets generated and analyzed in the current study are available upon editorial request.
